# The application of lung immune prognostic index in predicting the prognosis of 302 STS patients

**DOI:** 10.3389/fonc.2024.1460600

**Published:** 2024-09-09

**Authors:** Yong Jiang, Chang Zou, Xuanhong He, Longqing Li, Yi Luo, Minxun Lu, Zhuangzhuang Li, Taojun Gong, Yitian Wang, Li Min, Yong Zhou, Chongqi Tu

**Affiliations:** ^1^ Department of Orthopedics, Orthopedic Research Institute, West China Hospital, Sichuan University, Chengdu, China; ^2^ Model Worker and Craftsman Talent Innovation Workshop of Sichuan Province, West China Hospital, Sichuan University, Chengdu, Sichuan, China

**Keywords:** lung immune prognostic index (LIPI), soft tissue sarcoma, lactate dehydrogenase (LDH), derived neutrophil to lymphocyte ratio (dNLR), prognosis

## Abstract

**Background:**

Soft tissue sarcoma (STS) are heterogeneous and rare tumors, and few studies have explored predicting the prognosis of patients with STS. The Lung Immune Prognostic Index (LIPI), calculated based on baseline serum lactate dehydrogenase (LDH) and the derived neutrophils/(leukocytes minus neutrophils) ratio (dNLR), was considered effective in predicting the prognosis of patients with pulmonary cancer and other malignancies. However, the efficacy of the LIPI in predicting the prognosis of patients with STS remains unclear.

**Methods:**

This study retrospectively reviewed patients with STS admitted to our center from January 2016 to January 2021. Their hematological and clinical characteristics were collected and analyzed to construct the LIPI specific to STS. The correlations between various predictive factors and overall survival (OS) were examined using Kaplan–Meier and Cox regression analyses. Independent risk factors for OS were identified using univariate and multivariate analyses. Finally, a LIPI nomogram model for STS was established.

**Results:**

This study enrolled 302 patients with STS, of which 87 (28.9%), 162 (53.6%), and 53 (17.5%) were classified into three LIPI-based categories: good, moderate, and poor, respectively (P < 0.0001). The time-dependent operator curve showed that the LIPI had better prognostic predictive ability than other hematological and clinical characteristics. Univariate and multivariate analyses identified the Fédération Nationale des Centres de Lutte Contre le Cancer grade (FNCLCC/G), tumor size, and LIPI as independent risk factors. Finally, a nomogram was constructed by integrating the significant prognostic factors. Its C-index was 0.72, and the calibration curve indicated that it could accurately predict the three- and five-year OS of patients with STS. The decision and clinical impact curves also indicated that implementing this LIPI-nomogram could significantly benefit patients with STS.

**Conclusion:**

This study explored the efficacy of the LIPI in predicting the prognosis of 302 patients with STS, classifying them into three categories to evaluate the prognosis. It also reconstructed a LIPI-based nomogram to assist clinicians in predicting the three- and five-year OS of patients with STS, potentially enabling timely intervention and customized management.

## 1 Introduction

Soft tissue sarcoma (STS) is a rare and heterogeneous tumor mainly originating from the mesodermal layer (1). Its general occurrence rate is 4–5/100,000 individuals annually, with liposarcoma and leiomyosarcoma being the predominant subtypes (2). Extensive resection is the primary treatment method for early-stage STS. However, over 50% of patients with STS experience local recurrence or distant metastases after extensive resection (3), which contributes significantly to their reduced survival (4, 5). Consequently, the timely identification of high-risk factors for recurrence or distant metastasis in patients with STS is conducive to adjusting therapeutic strategies and disease counseling (6). Clinically, high-risk factors are mainly identified through tumor size, type, grading, and location. However, their identification depends greatly on clinicians’ experience and has a high false-positive rate. Therefore, an effective and straightforward prognostic predictive method is needed.

Several novel predictive biomarkers have recently been explored, including proteins, microRNAs (miRNAs), gene signatures, tumor-derived extracellular vesicles (EVs), and circulating tumor cells (CTCs) (7–14). However, the high cost and complexity of those techniques limit their further clinical application. Tumor-associated inflammation is an important factor in tumor development (15, 16). Multiple inflammation-related indicators, such as the neutrophil-to-lymphocyte ratio (NLR), platelet-to-lymphocyte ratio (PLR), lymphocyte-to-monocyte ratio (LMR), and serum lactate dehydrogenase (LDH), were found to be effective in predicting the overall survival (OS) of patients with lung cancer, gastric cancer, and pancreatic ductal adenocarcinoma (17–19). Lung Immune Prognostic Index (LIPI), calculated based on the baseline derived neutrophils/(leukocytes minus neutrophils) ratio (dNLR) and serum LDH, was found to be a valid prognostic indicator for malignancies treated with immune checkpoint inhibitors or chemotherapy (20–22). A LIPI and LIPI-related predictive model were also reported for osteosarcoma (23). However, to our knowledge, the efficacy of the LIPI in predicting the prognosis of patients with STS remains uncertain.

This study aimed to establish a LIPI for patients with STS and verify its prognostic significance. It first constructed and validated a LIPI for STS and then constructed a LIPI-based prognostic nomogram for patients with STS.

## 2 Patients and methods

### 2.1 Patients

**Figure 1:**
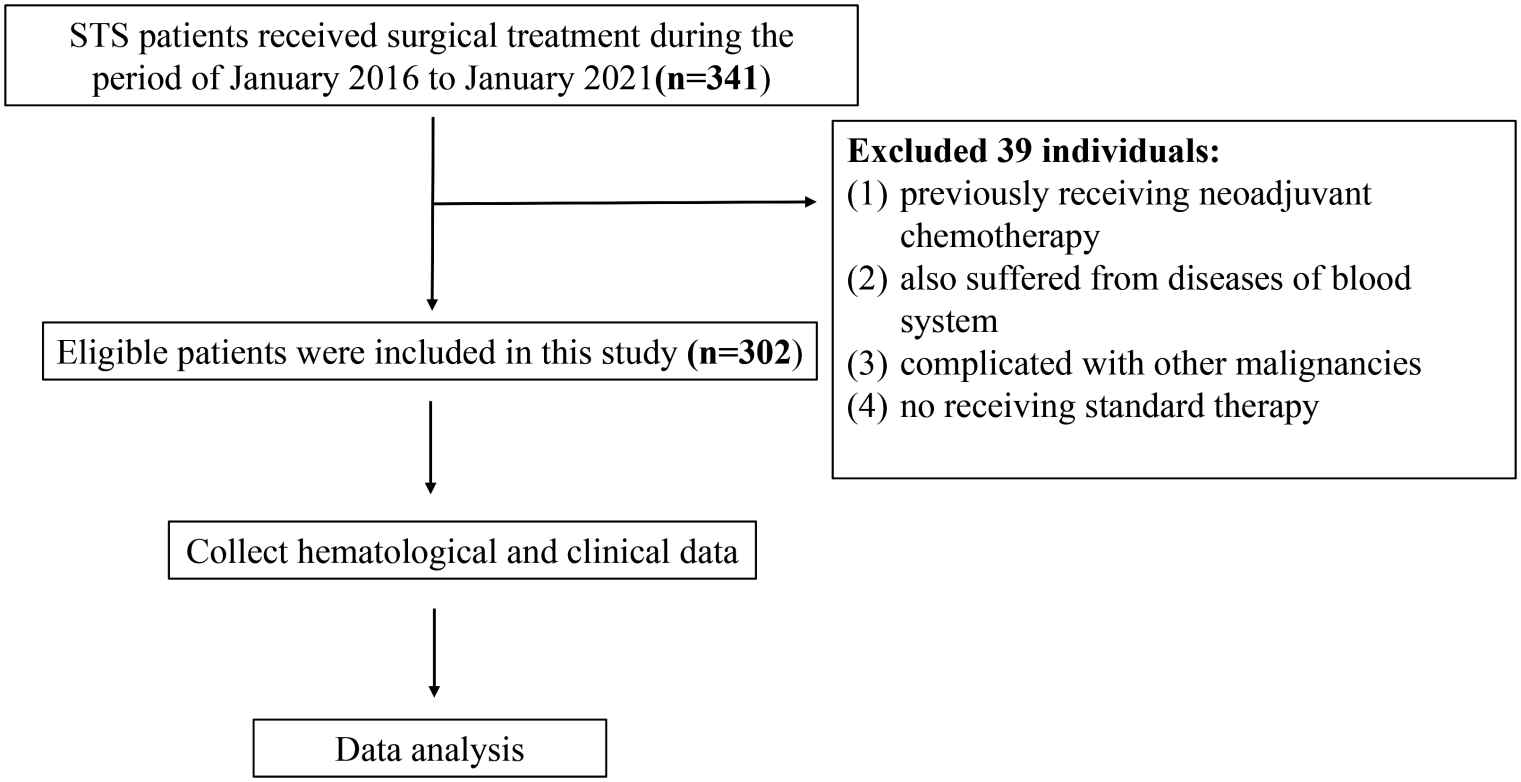
The flowchart for patient selection and study design.

STS patients admitted into the Musculoskeletal Tumor Center of West China Hospital during the period of January 2016 to January 2021 were reviewed. The inclusion criteria are as follow: 1. patients with pathology confirming the diagnosis of STS; 2. patients with complete hematological test results in our hospital; 3. patients who underwent standard treatment in our center. The exclusion criteria are as follow: 1. patients who had previously received neoadjuvant chemotherapy before the first consultation in our center; 2. patient also suffered from diseases of blood system; 3. patients complicated with other malignancies; 4. patients did not receive standard therapy. Eventually, there were in total of 302 patients included and each of them was followed up regularly until death or January 2021. The follow-up rules obeyed our previous studies (23). The flow chart of the study design is shown in **Figure 1**.

The ethics Committee of West China Hospital approved this study and each participant signed the written informed consent.

### 2.2 Data collection and analysis

Hematological markers, such as leukocyte count (Leut#), neutrophil count (Neut#), lymphocyte count (LYMPH#), platelet count (PLT), and lactate dehydrogenase (LDH), were acquired from the primary blood routine of 302 STS patients before neoadjuvant chemotherapy. The formulas for calculating the NLR, PLR, and dNLR are as follows: NLR = Neut#/LYMPH#, PLR = PLT/LYMPH#, and dNLR = Neut#/(Leut#-Neut#). Furthermore, clinical data including age, gender, tumor size, and tumor location, were collected and analyzed. The OS was calculated from the diagnosis date to the death date or the last follow-up date. The receiver operating curve (ROC) was applied to calculate the optimal cutoff value of each index and the hematological index was converted into binary variables.

### 2.3 Establishment and validation of the LIPI in 302 STS patients

dNLR is combined with LDH to establish LIPI for STS. Subsequently, the prognostic effect of LIPI, clinical features and other hematological variables on OS of STS was assessed. In order to determine if LIPI is an independent predictor of STS in patients, the univariate and multivariate analyses were conducted. Significant factors in univariate analyses were then included to multivariate analyses to explore the independent prognostic factors for STS patients.

### 2.4 Construction and evaluation of the LIPI-based nomogram for STS

After the above process, significant STS prognostic predictors were carefully selected and integrated to construct a nomogram. As shown, the total score for each STS patient was calculated by summing the scores of all factors. The nomogram presents the total points and the corresponding probability of OS. Harrell’s concordance index and calibration curve were respectively applied to estimate the discriminative ability and the extent of accuracy of the nomogram. The diagonal served as a reference line and represented the best forecast. To assess the clinical application of the nomogram and predict reduction intervention probability per 100 patients, the decision curve analysis (DCA) and the clinical impact curve was applied, respectively.

### 2.5 Statistical analysis

The Kolmogorov–Smirnov test was used to assess whether continuous variables were normally distributed, and the Mann–Whitney U test or Spearman correlation analysis was used to assess differences between continuous variables according to the results. The normal distribution of continuous variables and the differences between continuous variables were evaluated by Kolmogorov-Smirnov test and the Mann–Whitney U test or Spearman correlation analysis, respectively. Besides, the chi-square test and Fisher’s was performed to assessed the categorical variables based on the number of individuals in each group. R software, version 4.1.0 (Institute for Statistics and Mathematics, Vienna, Austria) was applied to perform the statistical analyses. P values < 0.05 were considered as statistically significant.

## 3 Results

### 3.1 Patient demographics and optimal cutoff values of hematological factors

**Figure 2:**
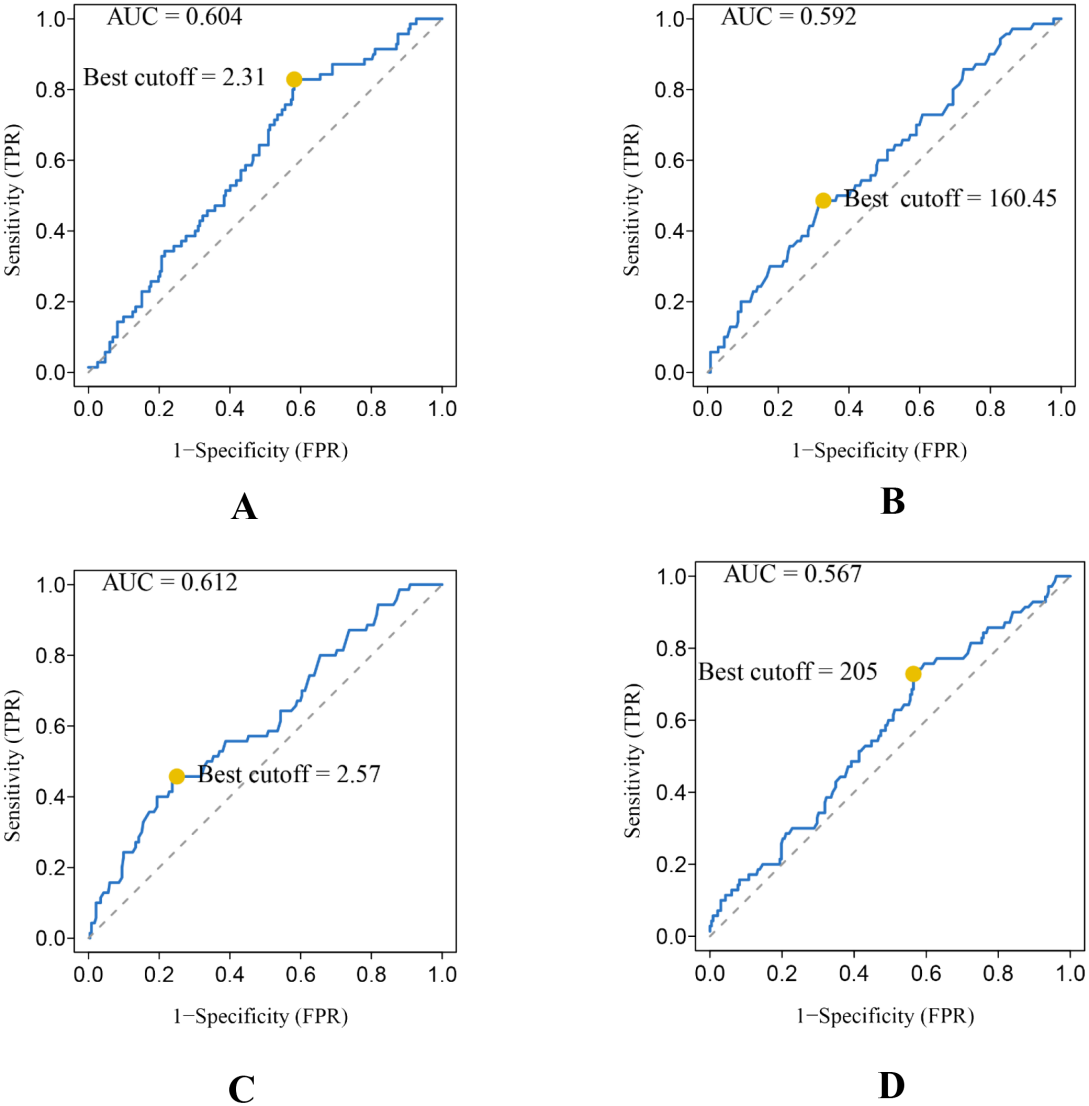
Conducting ROC analysis for various hematologic biomarkers. **(A–D)** The AUC and optimal cutoff values of NLR, PLR, dNLR, and LDH are as follows. Sensitivity is represented on the vertical axis, while 1-specificity is depicted on the horizontal axis.

A total of 166 males and 136 females were included. The average age of the 302 STS patients was 51.19 ± 18.58 years (ranging from 23 to 81 years). Tumors involved the limbs in 249 patients and extra-limbs in 53 patients. For tumor size, 34 patients had tumors smaller than 5cm, 151 patients had tumors larger than 5cm and smaller than 10cm, and 117 patients had tumors larger than 10cm (**Table 1**). A total of 70 patients died at the end of follow-up, and the median OS was 44.30 ± 26.97 months. The optimal cutoff values and AUC of NLR, PLR, dNLR, and LDH are 2.31mmol/L and 0.604, 160.45 mmol/L and 0.592, 2.57 mmol/L and 0.612, and 205 mmol/L and 0.567, respectively (**Figure 2**).

**Table 1:** Patients demographics.

	Patients	LIPI			P-value
		Good	Intermediate	Poor	
**Total patients**	302	87	162	53	
**Age (years)**	50.5	49.8	50.2	53.0	
**Gender**					0.870
Male	166	45	88	33	
Female	136	42	74	20	
**FNCLCC**					0.786
Stage 2	82	22	43	17	
Stage 3	220	65	119	36	
**Location**					0.081
Upper extremity	56	15	32	9	
Lower extremity	193	59	108	26	
Trunk	53	13	22	18	
**Tumor Size**					0.380
T<5 cm	34	2	21	2	
5 cm<T<10 cm	151	27	86	27	
T>10cm	117	48	55	48	

### 3.2 Establishment and validation of the LIPI in STS

**Figure 3:**
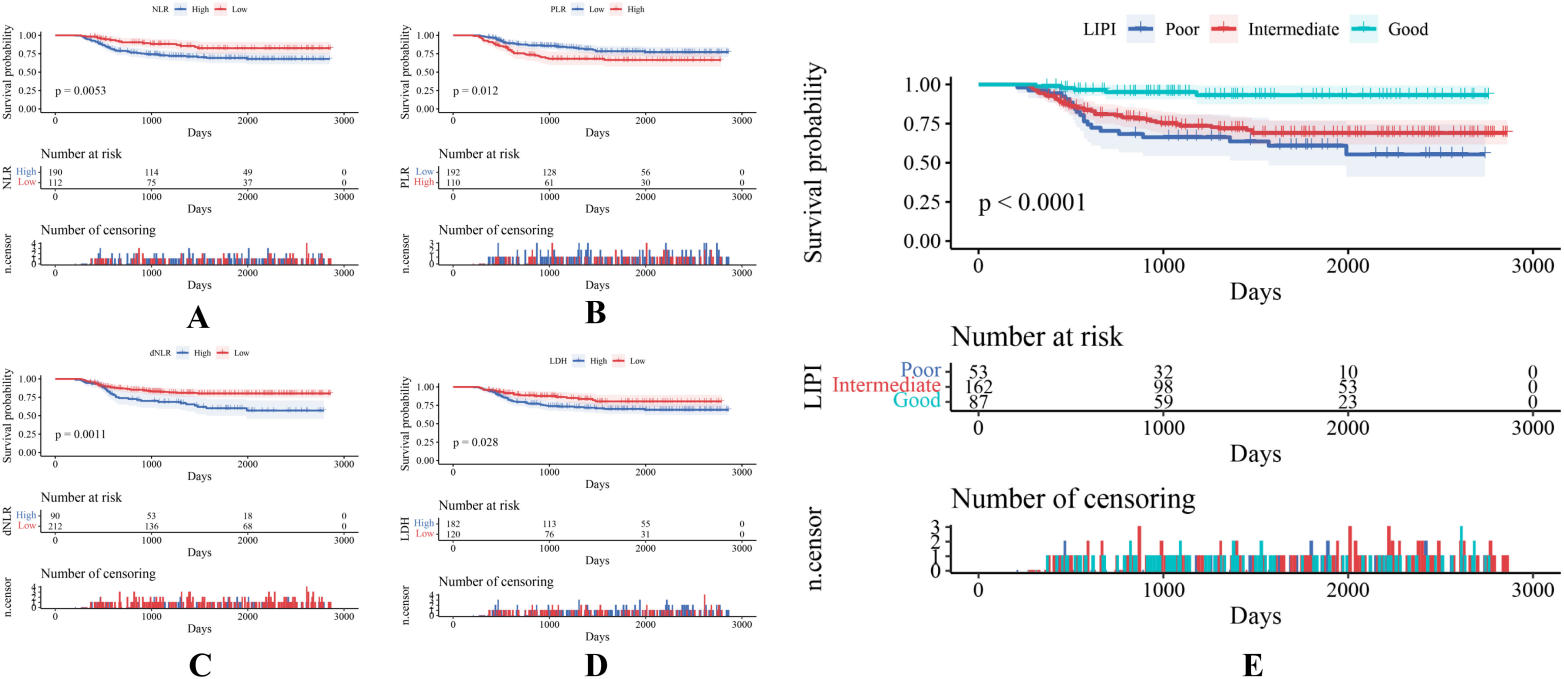
**(A–E)** Various hematological indexes of 302 patients with STS are reflected by KM survival curves.

302 STS patients were divided into different groups according to different hematological biomarkers. Compared with the high NLR score group, STS patients in the low NLR group demonstrated a better survival probability (P = 0.005) (**Figure 3A**). Compared with the high PLR score group, the STS patients in the low PLR group showed a better probability (P = 0.012) (**Figure 3B**). Compared with the high dNLR score group, STS patients in the low dNLR group showed a better survival probability (P = 0.001) (**Figure 3C**). Compared with the high LDH group, STS patients in the low LDH group showed a better survival probability (P = 0.028) (**Figure 3D**). Then, we constituted the LIPI combining LDH with dNLR according to Mezquita et al. (22). The LIPI divided 302 STS patients into three groups, with 87 patients in good LIPI group, 162 patients in intermediate LIPI group, and 53 patients in poor LIPI group (P < 0.0001) (**Figure 3E**). Take the STS patient with low dNLR and high LDH score for example, this patient was classified as poor LIPI and tend to have a worse survival probability.

**Figure 4:**
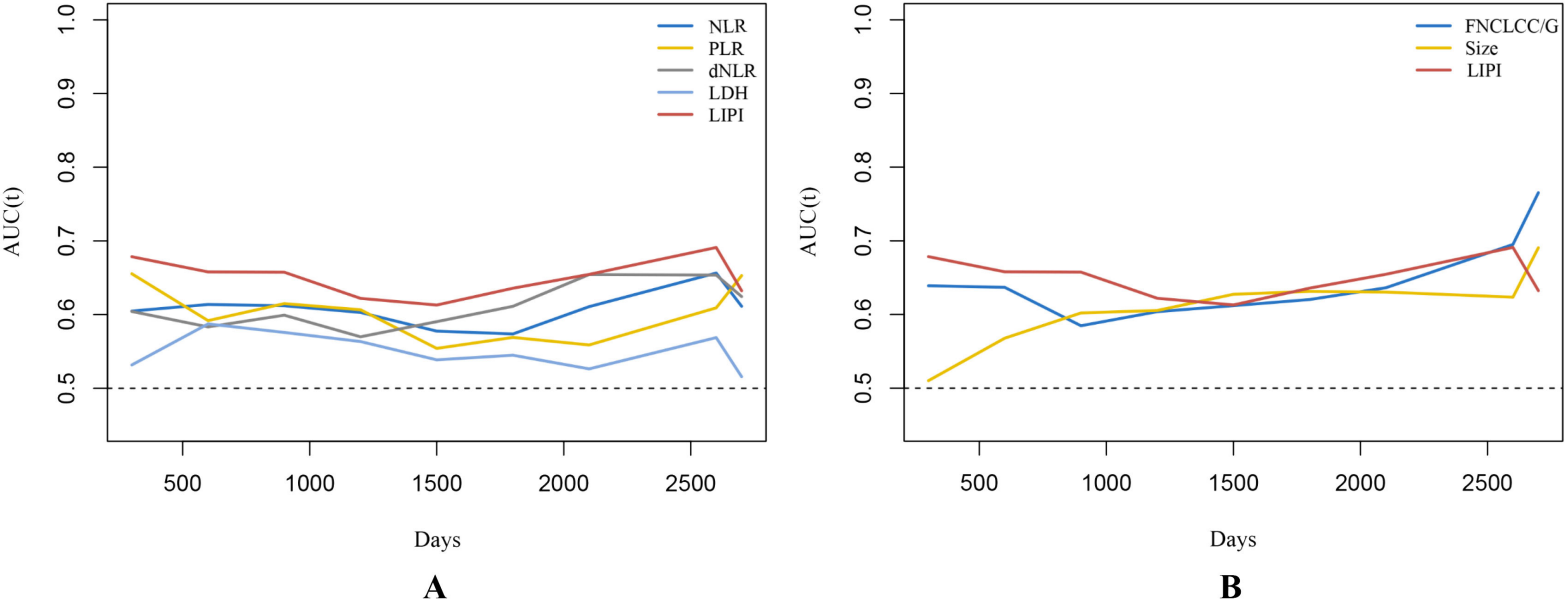
**(A)** Time-dependent ROC curves illustrate the variances in predictive capabilities of different hematologic markers. **(B)** The predictive abilities of STS independent prognostic factors are depicted by time-dependent ROC curves. Changes in predictive capabilities are reflected by time-dependent ROC curves, where a higher AUC value indicates superior predictive performance of STS.

As shown, a larger AUC in the LIPI than that of other single hematological markers including NLR, PLR, dNLR, and LDH was observed in the t-ROC curve (**Figure 4A**). Similarly, a larger AUC in the LIPI than that of clinical features was also demonstrated (**Figure 4B**).

### 3.3 Univariate analysis and multivariate analysis

**Figure 5:**
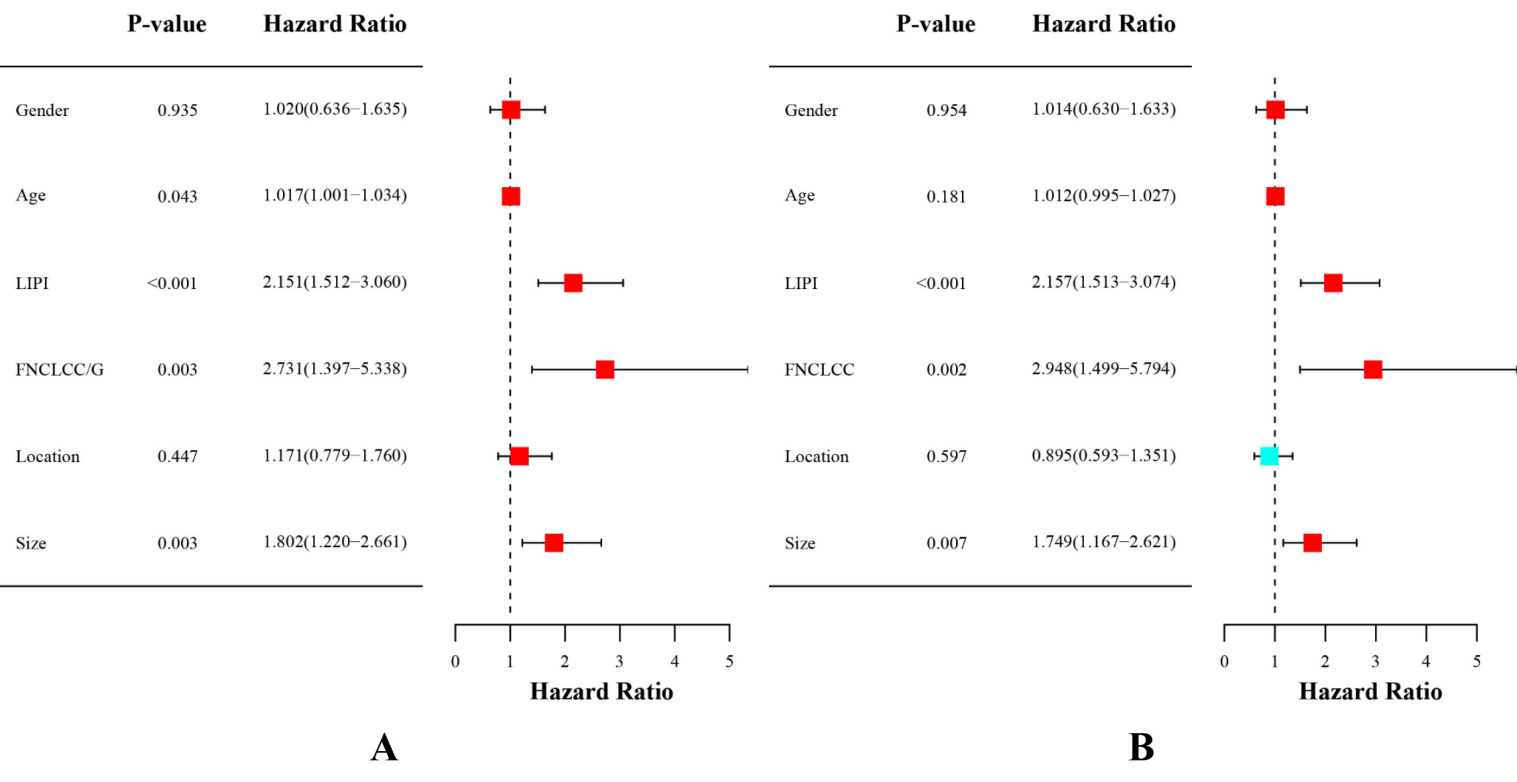
**(A)** Conducting univariate analysis for clinical characteristics and hematological biomarkers. **(B)** Conducting multivariate analysis for significant clinical characters and hematological biomarkers.

Univariate analysis and multivariate analysis were performed in 302 patients to further investigate the prognostic ability of variables in STS. The univariate analysis demonstrated the age (hazard ratio (HR)=1.017; [95% confidence interval CI] 1.001–1.034, P = 0.043), FNCLCC/G (HR =2.731 (1.397–5.338), P = 0.003), tumor size (HR=1.802 (1.220–2.661), P = 0.003) and LIPI (HR= 2.151(1.512–3.060), P < 0.001) were associated with OS (**Figure 5A**). Subsequently, multivariate analysis was performed to identify independent risk factors for OS. The multivariate analysis demonstrated that FNCLCC/G (HR=2.948 (1.499–5.794), P = 0.002), tumor size (HR=1.749 (1.167–2.621), P = 0.007), and LIPI (HR=2.157 (1.513–3.074), P < 0.001) were independent risk factors for STS (**Figure 5B**).

### 3.4 Construction and validation of LIPI-based nomogram

**Figure 6:**
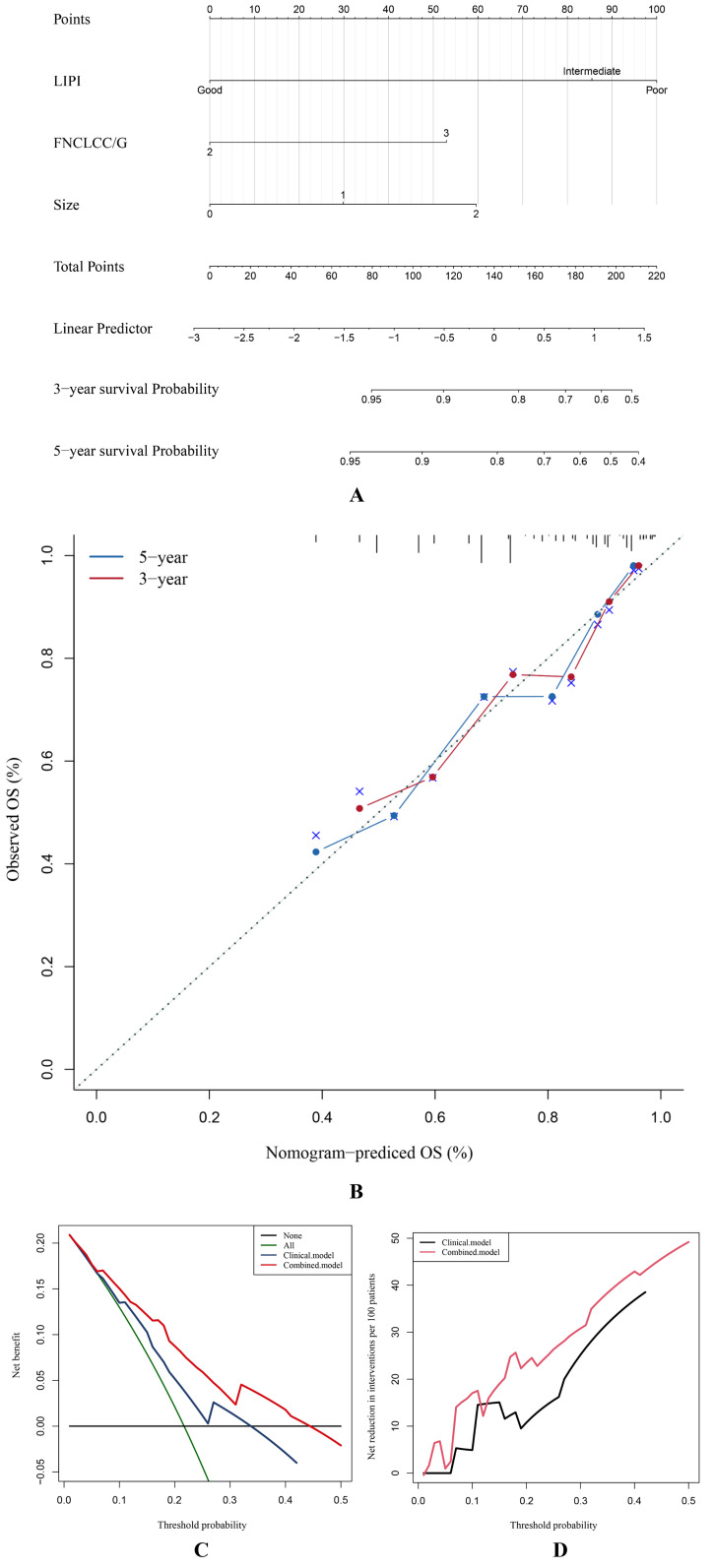
The STS overall survival nomogram of STS was constructed and validated. **(A)** LIPI, FNCLCC/G, and size are combined to construct the nomogram, and the total score of the nomogram was the sum of the scores of each covariate. **(B–D)** The calibration curve, decision curve analysis, and clinical impact curve verified the nomogram.

Finally, we constructed a nomogram combining the LIPI with clinical features to improve the clinical application of the LIPI. As shown, the Cox proportional hazards regression assigned a score based on the HR for each covariate, and the sum of the scores for each covariate was the nomogram total score (**Figure 6A**). The C-index of this STS nomogram was 0.72, and the calibration curve demonstrated that this nomogram could effectively predict the 3- and 5-year OS of STS patients (**Figure 6B**). Besides, we also explored the clinical benefits of this nomogram with clinical DCA (**Figures 6C, D**). Our results demonstrated that the addition of this nomogram with the LIPI could bring significant net benefits over the model with only clinical features.

## 4 Discussion

Our study retrospectively analyzed 302 patients with STS to identify indicators associated with STS prognosis and validate the predictive ability of LIPI preliminarily. The LIPI was an independent risk factor for predicting the prognosis of patients with STS. The LIPI had better prognostic ability than other indexes for patients with STS. A LIPI-based nomogram combining the LIPI and clinical features was also successfully constructed. It could effectively predict the three- and five-year survival of patients with STS. Our results indicate that the LIPI can be applied as a practical tool to predict the prognosis of patients with STS.

STS are a group of rare and heterogeneous tumors, accounting for 1% of all adult malignancies. Over 50% patients with STS experience local recurrence or distant metastasis after radical resection, greatly influencing their prognosis (24, 25). However, effectively predicting the prognosis of patients with STS is challenging due to the heterogeneity of STS and limited detection methods. Clinically, classic features such as the Enneking staging system, metastatic status, tumor location, histological type, and grade are used for prognostic evaluation (26). However, the evaluation of those clinical features depends highly on clinicians’ clinical experience, which may create false negatives. He et al. reported that the clinical features had a lower predictive ability than the Osteosarcoma Immune Prognostic Index (21). However, the tumor grade may be incorrect. Schneider et al. found that the rate of under-grading based on a core biopsy might reach up to 68% in leiomyosarcoma due to the need for neoadjuvant therapy (27). Moreover, 56% of retroperitoneal sarcoma tumors were inaccurately graded on biopsy compared to the pathological examination of the surgical specimen (28).

Recently, several novel prognostic factors, including proteins, miRNAs, gene signatures, tumor-derived EVs, and CTCs, have been reported to be effective in predicting the prognosis of patients with STS (29–34). Within clinical oncology, miRNAs have diagnostic, prognostic, and predictive importance and can serve as therapeutic targets (35). Advancements in RNA sequencing have enabled the development of transcriptomic signatures such as the Complexity INdex in SARComas (CINSARC), Genomic Grade Index (GGI), and hypoxia-associated signatures for STS prognosis (10). However, implementing single-cell RNA sequencing in regular clinical practice would be excessively expensive. In addition, it is unclear whether transcriptomic subgroups are retained during disease progression or treatment. Moreover, several studies have reported conflicting findings regarding the mutational status of CTCs compared to those of the matching original tissue or metastasis (36–38). Therefore, developing a straightforward, accurate, and low-cost prognostic model for STS is imperative.

Tumor-related inflammation is crucial in forming the tumor microenvironment (TME), and the relationship between STS and inflammation has been widely explored (15, 16). Several hematological factors have been reported to effectively predict the prognosis of patients with STS, including LDH, NLR, and so on (39–42). Lin et al. reported that preoperative serum LDH is an independent risk factor for OS in patients with undifferentiated pleomorphic sarcoma, with a high LDH level associated with poor prognosis (43). In addition, a high NLR was an independent risk indicator for poor prognosis in patients with STS (44). Moreover, a high PLR was significantly associated with decreased OS and was an independent risk factor for predicting clinical outcomes for patients with STS (45). However, those hematological indicators are single and inconclusive and may not reflect the inflammation status in the patients. Therefore, developing an index that can comprehensively evaluate the inflammation status *in vivo* may be a potential direction for predicting long-term OS.

Mezquita et al. introduced a comprehensive inflammation indicator, the LIPI, which is calculated based on the baseline dNLR and LDH and could assist in immunotherapeutic choices and predicting OS in patients with advanced pulmonary and extra-pulmonary malignancies (22, 23, 46). The efficacy of the LIPI in predicting prognoses has also been explored (21, 23). However, whether the LIPI could predict the prognoses of patients with STS has remained unclear. Therefore, this study initially investigated the correlation between the LIPI, also calculated based on baseline LDH and dNLR, and STS, and then developed a LIPI-based prognostic model for STS. The LIPI was better at predicting the long-term survival of patients with STS than the clinical markers tumor size and FNCLCC/G. Moreover, unlike other hematological indicators such as LDH, NLR, and dNLR, the LIPI could further divide patients into three levels, refining their prognosis risk stratification and guiding treatment selection. Furthermore, the t-ROC curves demonstrated that the LIPI had a better prognostic ability than other factors, indicating that this comprehensive index has more advantages than a single hematological inflammation index. Finally, the LIPI-based nomogram could help predict the OS of patients with STS and formulate treatment and follow-up strategies (**Figure 6**). For example, a patient with a LIPI of 210 has survival probabilities of about 50% and 40% at three and five years, respectively, and would, therefore, be expected to receive more frequent follow-up and positive intervention to improve their long-term survival. Based on the LIPI-based nomogram score of a given patient with STS, specific management measures and follow-up strategies could be arranged to realize personalized management strategies.

Research has demonstrated significant correlations between inflammation and all stages of development and malignant advancement of most types of cancer, as well as the effectiveness of anticancer therapies (47). Based on the Warburg effect, tumor cells have higher glucose intake and lactate production, one of the basic metabolic rewiring processes that occur during tumor malignant transformation (48). LDH is the key enzyme in anaerobic glycolysis, and the elevated serum LDH is a well-recognized predictor of poor survival in many types of tumors, including melanoma (49), osteosarcoma (50), and Ewing sarcoma (51). Tumor-associated neutrophils (TANs) accumulate in specific regions and can be activated by external stimuli from the TME, switching between anti- and pro-tumor phenotypes (52). Numerous studies have shown that tumor infiltrating lymphocytes can induce tumor cell apoptosis, influencing the immunotherapy effect and releasing cytokines, playing an important role in mediating chemotherapy and immunotherapy responses (53–55). In our study, the dNLR consisted of derived neutrophils and lymphocytes, which could reflect the systemic inflammation status in patients with STS to some extent. In addition, based on our study and previous studies, the dNLR could predict the prognosis of patients with STS better than the NLR. Because the dNLR contains more inflammatory indicators than NLR, it could better reflect the tumor-related inflammatory status, enabling better prognosis predictions for patients with STS (22). Similarly, Szkandera et al. reported a strong and independent correlation between high dNLR and poor OS in patients with STS (56). Our study indicates that patients with STS with an elevated serum dNLR (>2.57) tended to have a poor prognosis (**Figure 3C**). Therefore, the LIPI, combining the LDH and the dNLR, could represent tumor-related inflammation in patients with STS and could predict their prognosis.

However, our study had several limitations. Firstly, it was a single-center study and may be affected by bias. However, it included 302 patients with STS, making it the second-largest study specifically on patients with STS, and its results would be expected to reflect the role of LIPI in predicting the prognoses of patients with STS to a certain extent. Our future research will include a multicenter study that will further explore the efficacy of this prediction model in patients with STS. Secondly, this study was retrospective, which may introduce recall bias. However, prospective studies are difficult to conduct due to the rarity and heterogeneity of STS. Indeed, no prospective study has examined predicting the prognoses of patients with STS to date. Our future research will include multicenter and large-scale prospective studies to validate our findings.

## 5 Conclusion

This study explored the efficacy of the LIPI in predicting the prognosis of 302 patients with STS, classifying them into three categories to evaluate the prognosis. It also reconstructed a LIPI-based nomogram to assist clinicians in predicting the three- and five-year OS of patients with STS, potentially enabling timely intervention and customized management.

## Data Availability

The raw data supporting the conclusions of this article will be made available by the authors, without undue reservation.
